# Development of a competency for professional oral hygiene care of endotracheally-intubated patients in the intensive care unit: development and validity evidence

**DOI:** 10.1186/s12913-021-06755-z

**Published:** 2021-07-28

**Authors:** Eun-Sil Choi, Hie-Jin Noh, Won-Gyun Chung, So-Jung Mun

**Affiliations:** 1 Department of Dental Hygiene, The Graduate School, Yonsei University, Wonju, South Korea; 2 Department of Dental Hygiene, College of Software and Digital Healthcare Convergence, Yonsei University, 1 Yonseidae-gil, Wonju, Gangwondo 26493 Republic of Korea

**Keywords:** Intensive care units, Pneumonia, Ventilator-Associated, Oral hygiene, Dental hygienists, Dental staff, Clinical competence

## Abstract

**Background:**

Professional oral care in the intensive care unit may reduce the incidence of Ventilator Associated Pneumonia, which increases the patient’s mortality rate. This study aimed to develop a competency for professional oral hygiene care of endotracheally-intubated intensive-care patients.

**Methods:**

First, we developed a competency draft by reviewing the literature on oral hygiene care of patients in the intensive care unit. Next, we developed expert validity test questionnaires using this draft and conducted expert validity tests twice on 18 experts. We determined competency as a content validity index of 0.8 or more and received expert additive opinions about competency through an open-questionnaire expert validity test paper in this methodology study.

**Results:**

The content validity index ranged from 0.8 ~ 1.0 for all items. The competency of ‘professionalism’ comprised 2 sub-competencies with 7 behavioral indicators. ‘POHC preparation’ comprised 3 sub-competencies with 10 behavioral indicators. ‘POHC implementation’ comprised 3 sub-competencies with 6 behavioral indicators. ‘POHC evaluation’ comprised 2 sub-competencies with 8 behavioral indicators. Lastly ‘Cooperation among experts’ comprised 3 sub-competencies with 7 behavioral indicatiors.

**Conclusions:**

To provide patients with high quality oral hygiene care, these competencies should be implemented, and oral hygiene care professionals and related medical personnel should form a cooperative system.

## Background

Medical services and treatments provided in the intensive care unit (ICU) have a direct impact on patients’ lives and survival rates. Patients in the ICU, therefore, require sophisticated and well-informed management [1–3].

ICU patients exhibit compromised immunity [4] and are exposed to various pathogens, which lead to a high probability of secondary morbidity [5]. Moreover, mouth breathing and various medications can result in dryness of mouth (xerostomia), leading to compromised oral hygiene and secondary systemic infections, such as ventilator-associated pneumonia (VAP) caused by infection of the weakened oral tissue [6–8].

Between July 2014 and June 2015, we recorded 252 medical device-related infections in 169 different ICUs within 96 hospitals with ≥300 beds in South Korea. Among these, 735 (29.1%) were pneumonia cases and 443 of these pneumonia cases (60.3%) were VAP [9].

In ICU patients, VAP has been reported to prolong the duration of ICU admission, increase medical costs, and result in a two-fold increase in mortality [10].

The onset of VAP is associated with compromised oral hygiene [8, 11, 12]. The “ventilator bundle” concept introduced by the U.S. Institute for Healthcare Improvement promotes “daily oral care with chlorhexidine” as a proven method to reduce the onset of VAP [13, 14]. However, critically-ill patients experience difficulty in self-maintaining their oral hygiene due to unconsciousness or structural limitations of the ICU [7, 8, 15, 16].

Although ICU nurses in South Korea are aware of the importance of oral hygiene care and recognize that frequent care has a positive effect on oral hygiene, its maintenance is a relatively low-priority task [17]. A previous study that analyzed nurse distribution in adult ICUs of 422 medical institutions in South Korea reported that the average number of ICU patients assigned to one ICU nurse is four, which is more than twice the number of patients per ICU nurse in the US [18].

Another South Korean study reported that 53.3% of survey responders thought that dental hygienists should be responsible for oral hygiene care of patients in the ICU, while 44.4% responded that these tasks should be performed by nurses or nurse assistants, implying the need for a professional workforce that can ensure oral hygiene care in these patients [19]. In the U.S., some medical institutions hire professional dental hygienists to provide oral hygiene care in these patients to reduce the prevalence of VAP and limit medical costs [20].

Prendergast and Kleiman [20] suggested that if properly-trained oral hygiene experts provide professional oral hygiene care (POHC), the patients could benefit from greater health improvement at lower costs compared to the medical costs associated with treating the diseases caused by poor oral hygiene. In addition, international research has indicated that additional training and education are needed to improve techniques and knowledge for POHC in patients undergoing endotracheal intubation [21, 22].

The education required for POHC in ICU patients undergoing endotracheal intubation can be provided to dental professional via the process of competency development, which can offer the necessary training and provide structured guidelines [23].

Nonetheless, barely any guidelines for competency development for oral hygiene maintenance in ICU patients undergoing endotracheal intubation has been suggested or outlined by experts. Dental hygienists are experts in oral hygiene maintenance, who should thus be adequately competent to prevent VAP in such ICU patients. This study was therefore performed to develop a competency of dental hygienists for professional oral hygiene care of endotracheally-intubated intensive-care patients.

## Methods

### Development of competency draft questionnaire

#### Review of literature

The opinions of three experts, domestic and foreign academic papers, job descriptions, protocol of nurse in the intensive care unit at general hospitals and clinical practice guidelines were reviewed. Totally, 3459 academic papers were searched using Boolean search (AND and OR) for keywords including “inpatients”, “hospital patients”,” intensive care units”,” oral hygiene”,” mouth hygienes”, “practice guideline”, and “clinical competence” from PubMed, Embase, Web of science, RISS, and Google Scholar. Among these papers containing guidelines or competencies for oral care of intubated patients in the intensive care unit were selected and then, by excluding duplicate papers, those written in languages other than English or Korean, those not related to oral care for intubated patients in the intensive care unit, and those without detailed guidelines or competencies for oral care methods, two papers were finally included.

#### Competency preliminary question

The final preliminary questionnaire consisted of 57 questions, 8 of which were regarding ‘professional occupation’, 10 were ‘before POHC’, 12 were ‘during POHC’, 8 were ‘after POHC’, and 19 were regarding ‘cooperation’.

#### Competency scale

In order to assess the validity of the preliminary questions, we evaluated the validity of each item as ‘very appropriate’ (4), ‘appropriate’ (3), ‘not appropriate’ (2), and ‘not at appropriate’ (1) [24]. In addition, we added four open-ended questions to the questionnaire to gather the experts’ opinions about ‘items to be added’, ‘items to be corrected’, ‘items to be deleted’, and ‘other comments’.

### Content validity verification

We conducted the content validity test twice on experts using questionnaires that consisted of competency drafts.

As experts for validation, we selected doctors and nurses working in the ICU of a general hospital, dental hygienists and nurses with experience in providing oral hygiene care to endotracheally-intubated patients in the ICU, and dentists and dental hygienists with ample knowledge and experience of professional oral hygiene care. For the first expert validity test, we recruited a panel of 10 persons, including 5 dental hygienists, 3 nurses, 1 dentist, and 1 doctor [24]. The second expert validity test was conducted on the same expert group, but 2 nurses, 4 dentists, and 2 doctors were added to increase the number of representatives in each group. The second validity test was conducted 10 ~ 14 days after the first [24]. We collected each questionnaire after 1 week from the date of distribution.

All methods were carried out in accordance with relevant guidelines and regulations.

### Analysis methods

We used the SPSS Statistics software for Windows, Version 23.0 (SPSS ver 23.0, IBM Corp., Armonk, NY, USA) to analyze the collected data. The general characteristics of the experts were analyzed by descriptive statistics and frequency. The content validity was measured by the content validity index (CVI) and only the items with a validity score of 0.8 or higher were selected [24].

## Results

### Literature review

#### Defining the initial competency

In previous studies, POHC competency was determined by referring to the contents classified in the management period before, during, and after POHC [25]. The ethical knowledge was classified separately by the competence of ‘professionalism” according to the study by Bae et al. [26] The ‘collaborative’ capacity was determined by referring to the contents of previous studies that stated the importance of cooperation among oral care workers [27–29]. Finally, the initial capacities of dental hygienists for professional oral care of intubated patients in ICU were composed of ‘professionalism’, ‘before POHC’, ‘during POHC’, ‘after POHC’, and ‘cooperation’.

#### Initial competencies and initial behavior indicators

Competency consists of knowledge, skill, and attitude, all of which must be expressed in concrete ‘behavioral indicators’ [30] Based on the contents of the literature review, we found 8 indicators of ‘professionalism’, 10 indicators of ‘before POHC’, 10 indicators of ‘during POHC’, 8 indicators of ‘after POHC’, and 19 indicators of ‘cooperation’.

### Content validity verification

#### General characteristics of expert groups

Eighteen experts were included in this study: 5 dental hygienists (27.8%), 5 nurses (27.8%), 5 dentists (27.8%), and 3 doctors (16.7%). The average working experience of the expert group was approximately 11 years. Table 1 shows the general characteristics of the expert groups that participated in the expert validity study.

**Table 1:** General characteristics of the expert groups (N(%))

General characteristics		First test	Second test
**Gender**	Man	3(30.0%)	8(44.4%)
	Woman	7(70.0%)	10(55.6%)
**Type of profession**	Dental hygienist	5(50.0%)	5(27.8%)
	Nurse	3(30.0%)	5(27.8%)
	Dentist	1(10.0%)	5(27.8%)
	Doctor	1(10.0%)	3(16.7%)
**Work experience (years)**	Less than 6	4(40.0%)	10(56.0%)
	11 to 35 or less	6(60.0%)	8(44.8%)
**Oral care experience in hospital (years)**	Less than 1	6(60.0%)	10(55.5%)
	2 to 10 or less	4(40.0%)	8(44.8%)
**Average work experience (years)**		12.20	11.33

#### Results of the first expert validity test

We selected a question with a content validity of 0.8 or more [24] and collected the opinions of the experts using the open questions.

We modified the items that differed according to the patient’s state of consciousness or intubation and added the contents to decide whether to apply POHC according to the systemic condition. We ensured that the items were expressed in specific terms and deleted duplicate content.

In the first professional validity test, 7 indicators were classified as ‘professionalism’, 10 indicators as ‘before POHC’, 6 indicators as ‘during POHC’, 7 indicators as ‘after POHC’, and 8 indicators as ‘cooperation’. We finalized 5 competencies and 38 behavioral indicators.

#### Results of the second expert validity test

In the second expert validity test, we selected items with a content validity score of 0.8  more (Table 2) [24]and collected the opinions of the experts using the open questions.

**Table 2:** Second expert validity test results

Competency	Behavioral indicators		CVI^b^
**1. Professionalism** (7 → 7 indicators)	**Ethical behaviors**	1.1 Protects patients’ personal information.	1.00
		1.2 Assesses patient’s consciousness level and informs the patient of the maintenance if needed (such as the start and end of POHC^a^) .	1.00
		1.3 Sets regular times for POHC or acts in compliance with pre-set times.	0.94
		1.4 Reports to the person in charge in case the patient experiences problems.	0.89
	**Professional behaviors**	1.5 Receives adequate training in order to provide POHC.	0.94
		1.6 Explains the importance of POHC to the patients undergoing endotracheal intubation.	0.94
	**Infection control**	1.7 Has knowledge of sterilization and sanitization procedures to prevent cross-contamination and acts accordingly.	1.00
**2. Before POHC ** (10 → 10 indicators)	**Infection control**	2.1 Ensures hand hygiene.	1.00
		2.2 Has knowledge of personal protective equipment (such as gloves) and utilizes what is needed.	1.00
	**Patient condition**	2.3 Has knowledge of the patient’s date of admission and diagnosis and provides appropriate POHC based on the patient’s disease.	0.94
		2.4 Assesses the patient’s consciousness level and whether the patient is undergoing intubation.	1.00
		2.5 Evaluates the patient’s eligibility for POHC.	0.72
		2.6 Assesses if any taping on the intubation tube is loosened.	0.94
		2.7 Assesses the pressure within the intubation tube and reports to the person in charge if there is an issue.	0.83
	**Preparation for practical work**	2.8 Evaluates oral hygiene status and documents the examination outcomes.	1.00
		2.9 Prepares the tools and equipment required for each individual patient at the time of POHC.	0.94
		2.10 Turns on the aspirator and connects the disposable suction tube to the aspirator catheter.	1.00
**3. During POHC** (6 → 6 indicators)	**Infection control**	3.1 Performs the necessary infection control during POHC (such as hand hygiene, etc.).	0.94
	**Patient condition**	3.2 Has knowledge of comfortable postures for the patient to safely undergo POHC.	0.89
	**POHC**	3.3 Has knowledge of POHC for ICU^c^ patients and performs accordingly.	0.94
		3.4 Uses the appropriate necessary medication based on the patient’s condition (e.g., chlorhexidine mouthwash, gel to relieve mouth dryness, etc.).	0.94
		3.5 Removes saliva or solution used for POHC using an aspirator or gauze.	1.00
	**Documentation**	3.6 Documents the performed procedures.	0.89
**4. After POHC ** (7 →8 indicators)	**POHC and organization**	4.1 Assesses potential residual solution or saliva in the oral cavity and removes it if needed after the procedure.	1.00
		4.2 Documents the amount, type, and color of the saliva covering the tools (e.g., toothbrush) or disposables used for maintenance.	0.89
		4.3 Cleans the equipment properly and disposes of the used materials and solutions appropriately.	1.00
	**Patient condition**	4.4 Confirms the location (or position) of the intubation tube.	0.94
		4.5 Confirms the safety of the patient’s posture after POHC, according to the patient’s systemic condition.	0.94
	**Preparation for next POHC**	4.6 Appropriately cleans, sterilizes, sanitizes, and stores the used equipment.	0.94
		4.7 Verifies the equipment stock and prepares the items required for the next POHC.	0.89
**5. Cooperation** (8 → 7 indicators)	**Professional behaviors**	5.1 Understands and applies the rules and regulations of the team and takes responsibility for their own actions within the team.	1.00
		5.2 Puts active effort into achieving the common goals of the team.	1.00
	**Problem-solving abilities**	5.3 Observes the performance of the teammates and corrects mistakes if needed.	0.89
		5.4 Predicts the needs of the teammates and provides help if needed.	1.00
		5.5 Respects the teammates and puts effort into understanding their situation and position.	1.00
		5.6 Asks for the teammates’ help when feeling overwhelmed by an excessive workload.	1.00
	**Communication**	5.7 Communicates with fluency using official language/vocabulary.	1.00
		5.8 Cooperates with oral hygiene management team members or other medical staff to provide optimal POHC.	1.00

^a^professional oral hygiene care, ^b^content validity index, ^c^intensive care unit

We discussed the scope of a dental hygienist’s work in Korea and assessed the viscosity and color of saliva. We felt the need for a system to record the POHC and oral status of patients to communicate effectively with nurses.

After the second validity test, the experts were classified as 7 indicators of ‘professionalism’, 10 indicators of ‘preparation for POHC’, 6 indicators of ‘performing POHC’, 8 indicators of ‘evaluation of POHC’, and 7 indicators of ‘expert cooperation’. We completed competency tools with 5 competency groups, 13 sub-competencies, and 38 performance indicators.

Table 3 shows the final competencies and definitions determined from the second expert content validity test of this study.

**Table 3:** Defining the final competency

Final Competency	Definition
**Professionalism**	The ethical and professional behavior demonstrated by the dental hygienist towards endotracheally intubated patients during all stages of POHC^a^ in the intensive care unit.
**Preparation for POHC**	Competency required before POHC
**Performing POHC**	Competency required during POHC
**Evaluation of POHC**	Competency required after POHC
**Cooperation among experts**	Cooperation with fellow dental hygienist and with other medical staff.

^a^ professional oral hygiene care

#### Correcting the vocabulary

After verifying the second experts’ validity, we completed the Korean version of the competency tool by evaluating the correctness of the first Korean vocabulary and the overall sentence flow [31]. After that, we translated the Korean version of the competency tool into the English version.

#### Final competency development

The competency of ‘professionalism’ consisted of 2 sub-competencies with 7 behavioral indicators. ‘Preparation for POHC’ competency consisted of 3 sub-competencies with 10 behavioral indicators. ‘Performing POHC’ competency consisted of 3 sub-competencies with 6 behavioral indicators. ‘Evaluation of POHC’ competency consisted of 2 sub-competencies with 8 behavioral indicators. Lastly, ‘Cooperation among experts’ competency consisted of 3 sub-competencies with 7 behavioral indicators. There were 13 sub-competencies and 38 behavioral indicators in 5 competency groups (Table 4).

**Table 4:** A dental hygienist competencies for POHC of endotracheally intubated patients in the intensive care unit

Competency	Subcategories of competency	Behavioral indicators
**1. Professionalism** (7 indicator)	**Ethical behaviors**	1.1 Protects patients’ personal information.
		1.2 Assesses the patient’s consciousness level and informs the patient of the content of maintenance if they are conscious (e.g., the beginning and end of POHC^a^)
		1.3 Reports to the medical staff in charge (physician or nurse) if the patient exhibits problems.
	**Professional behaviors**	1.4 Sets regular times for POHC or acts in compliance with pre-set times.
		1.5 Actively participates in the training to provide POHC.
		1.6 Understands the purpose and importance of POHC in intubated patients and is able to explain these to the caregiver if needed.
		1.7 Has knowledge of sterilization and sanitization processes in order to prevent cross-contamination.
**2. Preparation for POHC** (10 indicator)	**Performing infection control**	2.1 Ensures appropriate hand hygiene.
		2.2 Has knowledge of the required personal protective equipment (e.g., gloves, mask, eye shield, protective clothes, etc.) and prepares accurately.
	**Patient preparation**	2.3 Confirms the patient’s date of admission and diagnosis and evaluates the patient’s eligibility for POHC after consulting with the physician in charge.
		2.4 Assesses the patient’s consciousness level and endotracheal intubation status.
		2.5 Requests the medical staff in charge to assess the position of the endotracheal tube.
		2.6 Requests the medical staff in charge to assess the cuff pressure of the endotracheal tube.
		2.7 Reports to the medical staff in charge when abnormalities are found in the oral cavity and provides appropriate treatment.
	**Preparation for POHC**	2.8 Examines the patient’s oral condition (e.g., oral tissue, saliva, etc.) and enters the examination outcomes into the recording sheet.
		2.9 Prepares the tools and equipment required for each individual patient at the time of POHC.
		2.10 Turns on the aspirator and connects the disposable suction tube to the aspirator.
**3. Performing POHC** (6 indicator)	**Performing infection control**	3.1 Performs the necessary infection control during POHC (e.g., ensuring hand hygiene, wearing personal protective equipment, etc.).
	**Assessing patient condition**	3.2 Consults with the medical staff in charge to ensure a safe and comfortable position for the patient while undergoing POHC.
	**Performing** **POHC**	3.3 Has knowledge of the normal range of oxygen saturation levels and reports to the medical staff in charge if abnormal levels are observed during POHC.
		3.4 Has knowledge of POHC for ICU patients undergoing endotracheal intubation and utilizes the appropriate equipment for POHC.
		3.5 Uses the appropriate necessary medication based on the patient’s condition (e.g., chlorhexidine, gel to relieve mouth dryness, etc.)
		3.6 Removes saliva or solution used for POHC using an aspirator or gauze.
**4. Evaluation of POHC** (8 indicator)	**Organizing POHC equipment and materials**	4.1 Assesses potential residual solution or saliva in the oral cavity after POHC and removes it if needed.
		4.2 Enters the performed procedures and outcomes (i.e., notes on patient condition, type of saliva, etc.) in the recording sheet.
		4.3 Cleans the equipment properly and disposes of all materials and solutions appropriately.
		4.4 Appropriately cleans, sterilizes, sanitizes, and stores the used equipment.
		4.5 Prepares the items required for the next POHC in advance.
	**Assessing patient condition**	4.6 Asks the medical staff in charge to confirm the appropriate positioning of the endotracheal intubation after management.
		4.7 Confirms the safety of the patient’s posture after POHC and asks for confirmation from the medical staff in charge.
		4.8 Assesses the patient’s condition and communicates with the medical staff in charge to transfer the patient to receive additional dental treatment if necessary.
**5. Cooperation among experts** (7 indicator)	**Professional behaviors**	5.1 Follows the regulations and takes responsibility for their own actions within the team.
		5.2 Puts active effort into achieving the common goals of the team.
	**Problem-solving abilities**	5.3 Observes the performance of the teammates and assesses improvements that need to be made.
		5.4 Asks for help or provides help to teammates if needed.
		5.5 Respects the teammates and puts effort into understanding their situation and position.
	**Communication**	5.6 Communicates with fluency using official language/vocabulary.
		5.7 Cooperates with the POHC team members or other medical staff to provide optimal oral hygiene management service.

^a^ professional oral hygiene care

## Discussion

In this study, we developed a competency tool for dental hygienists, which is needed to prevent VAP and provide POHC for patients undergoing endotracheal intubation.

In such cases, it is important to assess the admission date of the patient. Risk of VAP increases by 1% per day during mechanical ventilation [32] and VAP can occur in critically-ill patients who undergo mechanical ventilation for 48 hours or longer [1].Patients undergoing endotracheal intubation show 6- to 21-fold increase in VAP morbidity than patients without intubation [1, 33]. Therefore, oral care is critical for such patients in order to reduce the risk for pneumonia.

Endotracheal tubes (ETT) lead to increased complexity within the oral cavity and thereby hinder oral assessment [3, 8, 34]. In addition, there is a risk of the tube being detached during repositioning, which makes POHC difficult [15]. Since patients undergoing endotracheal intubation require their mouths open for longer periods, bacteria or detached epithelial cells may pile up. Furthermore, in the case of oral edema, the ETT may exert pressure on the oral cavity and worsen the patient’s oral condition. Therefore, the ETT needs to be cleaned, maintained, and regularly repositioned during the POHC process [35].

As various diseases or intracranial lesions may affect the consciousness level of a patient, it is important to accurately assess the consciousness level of each patient to evaluate their oral condition [2, 16, 36]. Unconscious patients or patients with decreased levels of consciousness often keep their mouths open and constant mouth respiration makes their oral mucosa more susceptible to dryness [37]. Moreover, unconscious patients often undergo intubation feeding, resulting in reduced saliva secretion and consequently reduced activity of the salivary glands [7].

Dental hygienists need to cooperate with other medical staff to understand whether a patient will be eligible for POHC, based on the patient’s overall condition. In short, they need to comprehensively examine the patient’s general physical condition and oral state to confirm whether the patient is ready for POHC. Systemic medical treatment in ICU patients can make POHC difficult [3, 15], which makes it essential to understand the relationship between the patient’s overall condition and their oral health status [38].

For appropriate oral care, accurate assessment of the patient’s oral state is crucial [39]. Understanding a patient’s oral condition is essential to assess and confirm that the patient indeed needs oral care [37]. Furthermore, as patients in the ICU cannot express or manage their oral state, it is the responsibility of the dental hygienists to record the oral health condition of such patients [15]. According to a previous study by Choi et al., 67.3% nurses perform oral assessment but only 12.8% utilize the assessment tool to document the outcomes [17].

One of the most severe side effects of ventilator-based treatment is hypoxia during aspiration, which can be identified via oxygen saturation levels [40]. Decreased oxygen saturation in addition to purulent expectoration, changes in white blood cell levels, and diagnostic findings on chest radiographs indicate pneumonia [41]; therefore, oxygen saturation should be assessed during POHC.

Among the additional comments received, the opinions shared by all groups were related to the scope of work. Based on the current dental hygienist scope of work in Korea [42], the responsibilities of a dental hygienist do not include inspecting the intubation and checking the patient’s position or the cuff pressure during intubation. Therefore, it is necessary for the medical staff, such as nurses and doctors, to verify the above factors and cooperate with the dental hygienist.

The finalized competency list comprised of five sections, including one named “cooperation among experts,” as cooperation among experienced individuals with various levels of expertise can lead to better treatment outcomes and reduced medical costs [20]. Patients must be provided with a comprehensive and professional medical service including oral care, and to this end, a system for multidisciplinary cooperation needs to be developed.

In Japan, there is a specialized system for pre- and post-operative oral hygiene care for ICU patients, which involves inspection by a team of medical experts, including dental hygienists, physicians, nurses, nutritionists, and pharmacists. This team builds on the expertise of its members to provide comfort to the patient prior to the surgery. All costs associated with the establishment and management of this system are covered by national health insurance. Apart from this system, there are about 40 items in separate categories of medical management that are covered by dental health insurance [43].

Studies assessing the competency of the workforce involved in expert-level oral hygiene care in ICU patients undergoing endotracheal intubation are rare, except for two studies [22, 25] assessing nurses in Jordan. These studies were limited in addressing the actual practice of oral care, as they developed competencies from nurses’ perspectives. The novelty of the present study is that it determines a detailed and comprehensive competency tool that reflects the opinions of dental and medical professionals.

In practical terms, it was difficult to recruit study participants with an experience of providing oral care in the ICU, but we tried to increase the validity of the competency tool by having the expert validity established by professionals with an average of 11 years of in-hospital oral care experience in each field.

Based on the competencies presented in this study, multilateral efforts are needed to develop an oral hygiene management system for patients undergoing endotracheal intubation. Relevant education programs must be developed to train competent dental hygienists, and legal systems need to be developed to ensure the provision of oral care for in-patients. Furthermore, future studies need to explore practical solutions to the problems in implementing such systems in hospitals.

Any future study could be strengthened by including perceived competencies of POHC from patients’ and their carers’ perspectives. Additionally future studies on the success of any POHC intervention could include assays of the oral microbiome before and after the intervention, as well as looking at reduction in VAP.

In addition, using the capabilities developed in this study, a study to determine the differences between different occupations related to oral care of endotracheally-intubated patients in the intensive care unit can be conducted in the future.

## Conclusions

The 5 competencies, 13 sub-competencies, and 38 behavioral indicators, developed in this study, would be able to evaluate the competency of dental hygienists for POHC of intubated patients. To provide patients with high quality oral hygiene care, these competencies should be implemented, and oral hygiene care professionals and related medical personnel should form a cooperative system.

## Data Availability

The datasets used and/or analysed during the current study are available from the corresponding author on reasonable request.

## Abbreviations

ICU: Intensive Care Unit
VAP: Ventilator-Associated Pneumonia
POHC: Professional Oral Hygiene Care
CVI: Content Validity Index
ETT: Endotracheal Tubes
